# Intravitreal infusion: A novel approach for intraocular drug delivery

**DOI:** 10.1038/srep37676

**Published:** 2016-11-25

**Authors:** Jiao Tian, Jia Liu, Xiao Liu, Yangyan Xiao, Luosheng Tang

**Affiliations:** 1 Department of Ophthalmology and Centre of Eye Research, The Second Xiangya Hospital, Central South University, Changsha, China

## Abstract

Intraocular injection has become an increasingly important intervention in the treatment of posterior segment diseases. However, an acute intraocular pressure (IOP) elevation after intravitreal injection is a common concern. This study aimed to evaluate the efficacy of intravitreal infusion in maintaining stable IOP in a rabbit model. Trypan blue (TB) 0.06% with an external pump was used to evaluate intravitreal infusion in rabbit eyes. Groups A (50 μL), B (100 μL), C (150 μL), and D (200 μL) were slowly infused over 30 minutes with TB. As a control, Group E underwent conventional intravitreal injection of 100 μL of TB. Group F received a bolus infusion of 100 μL of TB within 1 minute. The mean increases in IOP during infusion for each group were: Group A (7.93 ± 3.80 mmHg), B (13.97 ± 3.17 mmHg), C (19.91 ± 6.06 mmHg) and D (29.38 ± 8.97 mmHg). Immediately post-injection in group E the mean increase in IOP amounted to 34.33 ± 6.57 mmHg. The mean increase in IOP of group F after bolus infusion was 49.89 ± 1.71 mmHg. Intravitreal infusion maintains a stable IOP and provides a controlled infusion speed compared with intravitreal injection.

Intravitreal injection is the currently accepted method to treat posterior segment diseases, such as age-related macular degeneration (AMD), diabetic retinopathy, vascular occlusions, cystoid macular edema, uveitis, viral retinitis, endopthalmitis, and retinal detachment^1^,^2^,^3^. This procedure allows a direct application of the drug into the posterior eye, thus eliminating the barriers that are common with topical and systemic administration. Also, a higher intraocular bioavailability in posterior segment tissues yield the more efficacious treatment of posterior eye diseases^2^,^4^.

In recent years, numerous studies have reported an acute volume-related intraocular pressure (IOP) elevation after intraocular injections^5^,^6^,^7^,^8^,^9^,^10^,^11^,^12^,^13^,^14^,^15^. Lemos-Reis *et al*. reported postoperative IOPs greater than 50 mmHg in 32% of patients who received intravitreal 50 μL bevacizumab injections^6^. In two clinical studies, the immediately post-injection mean IOP respectively rose to 53.9 ± 18.2 mmHg^10^ and 57.9 ± 11.4 mmHg^12^ after volumes of 100 μL injected into vitreous cavity. A transient increase in IOP can lead to ischemia of the optic nerve head and retina in response a decrease in perfusion pressure^16^,^17^,^18^,^19^. In most cases direct visualization of the optic nerve perfusion might be enough, but in patients with pre-existing glaucomatous damages or a history of vascular diseases, intravitreal injection should be seriously considered because those eyes may be more vulnerable to the effects of increased IOP^10^,^12^,^14^,^20^. Several studies report that patients with ocular and systemic ischemia have acute vision loss that is associated with retinal artery occlusion after an intravitreal injection of bevacizumab^21^,^22^,^23^. Post-injection subconjunctival reflux leads to a decrease in IOP^5^,^6^,^24^. Reflux and subconjunctival bleb formation has been reported following intravitreal drug delivery^24^,^25^,^26^. The bleb may be composed of liquefied vitreous or reflux of the injected drug. Drug reflux through the injection site may lead to the inaccuracy of the drug doses and limit the therapeutic efficacy of medications delivered by intravitreal injection^25^,^26^. The eye is a closed system and is sensitive to an increase of even small volumes of solution. It is characterized by IOP elevation and/or drug and vitreous reflux after intravitreal injection.

There are several reports that mention methods to prevent an increase in IOP after intravitreal injection. Routine prophylactic use of IOP-lowering medications is essentially ineffective in preventing IOP spikes after intravitreal injection^9^. Prophylactic anterior chamber paracentesis decreases the incidence of high postoperative IOP spike^8^. However, fluctuations in the IOP are greater with intravitreal injection immediately after anterior chamber paracentesis. Moreover, there are other complications with anterior chamber paracentesis, including lens damage, hyphema, patient discomfort and infection.

Though these IOP spikes after injection are common and no ideal method has been established to deal with them so far, the duration is thought to be transient. More than 95% of IOP values are under 30 mmHg in 30 minutes after intravitreal injection^7^,^8^,^9^,^10^,^11^,^15^. Robust IOP homeostatic mechanisms keeps ocular pressures within relatively narrow acceptable bounds throughout most peoples’ lives^27^. This suggests that a fast and efficient IOP homeostatic mechanism is employed following the increase in volume of the vitreous cavity after injection. A more effective treatment of posterior segment diseases may be a continuous, chronic drug delivery method that maintains a stable IOP.

A novel therapeutic strategy for continuous drug delivery is a posterior implantable drug pump. This has been demonstrated to effectively deliver microdoses when used in animal models^28^,^29^ and in a small cohort of diabetic macular edema patients^30^. Recently, a phase 2 study evaluated the efficacy of microvolume injections or infusions of brolucizumab versus ranibizumab in previously untreated patients with neovascular AMD. The microvolume infusions were administered over a 16-minute period via an external pump connected to a cannula placed in the patient’s eye^31^ and demonstrated that intravitreal infusion is a potential therapy in treating diseases of the posterior segment.

To further evaluate this procedure, we designed this study using a pump to perform an intravitreal infusion in different infusion volumes and compared the IOP with the intravitreal injection model in experimental animals.

## Results

### Experimental Design

Rabbits were fitted with insulin pumps that were used as intravitreal infusion devices. The experiment was designed with four groups with different volumes to evaluate the safety of infusion speed. Groups A (50 μl), B (100 μl), C (150 μl) and D (200 μl) were slowly infused over 30 minutes with Trypan blue (TB) using an insulin pump. Each group included six rabbits and infusions were done in the right eye. In order to compare to standard intravitreal injections two additional experimental groups were included. According to the results of group A–D, group B was set as the reference group. Group E received a bolus injection of 100 μL of TB, similar to the clinical procedure performed in humans. Group F received a bolus infusion with 100 μL of TB within one minute using an infusion pump and the needle was removed after 30 minutes.

### Intraocular Pressure (IOP)

**Figure 1:**
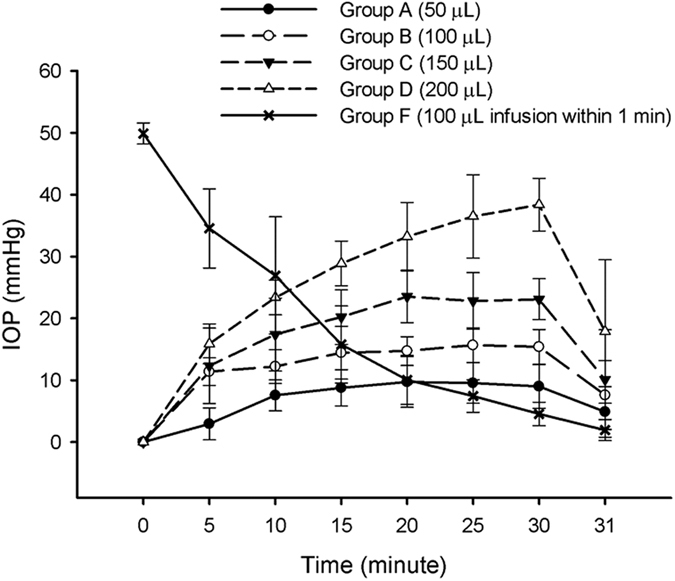
Measurement of IOP in experimental groups. Intraocular pressure (IOP) with standard deviation in the infusion groups (**A**–**D**,**F**).

Baseline IOP (mean ± standard deviation) was 10.40 ± 1.67 mmHg. The mean increases in IOP during infusion for each group were: Group A (7.93 ± 3.80 mmHg), B (13.97 ± 3.17 mmHg), C (19.91 ± 6.06 mmHg) and D (29.38 ± 8.97 mmHg). As seen Fig. 1, infusion of higher volumes of TB significantly increased the IOP in groups A-D. Multivariate analysis of variance (MANOVA) indicated a statistically significance increase in the IOP in groups A–D (*p* < 0.001). The mean increases in IOP immediately after removal of the needle in each group was A (4.89 ± 4.09 mmHg), B (7.61 ± 5.58 mmHg), C (10.11 ± 8.05 mmHg), and D (17.89 ± 11.61 mmHg). Immediately post-injection in group E the mean increase in IOP compared with preoperative values amounted to 34.33 ± 6.57 mmHg. The IOP values in group F after bolus infusion were all more than 60 mmHg and they were all recorded as 60 mmHg. Compared with baseline the increase in IOP was 49.89 ± 1.71 mmHg. The IOP decreased rapidly over the next 30 min (Fig. 1) and the mean IOP at 30 min before removal of the needle was 14.67 ± 2.46 mmHg, which was similar to the reported IOP observed 30 minutes after intravitreal injection^7^,^8^,^9^,^10^,^11^,^15^.

### Reflux

IOP measurements and reflux scores showed a significant degree of correlation (r = 0.71). The mean IOP values prior to removal of needle (at 30 minutes) directly correlated to reflux scores in all infusion eyes.

### Residual volume

No difference was observed between the predicted residual volume and the actual volume remaining in the reservoir in all infusion groups. The residual volume after intravitreal injection in group E was approximately 10 to 40 μL.

### Eye changes

The anterior chamber was observed to become shallower and the cornea became less transparent at the moment of injection in group E and bolus infusion in group F. Light to moderate corneal edema was observed in several animals in group C and all animals in group D. No adverse events were noted during the post-operative observation in any group.

## Discussion

As the intravitreal injection of therapeutic medication plays an increasingly larger role in ophthalmology, its implementation continues to be modified and refined. An acute volume-related IOP elevation occurs after intraocular injection^5^,^6^,^7^,^8^,^9^,^10^,^11^,^12^,^13^,^15^. In addition to the effect of doses of the injected drug, the IOP is also affected by other factors, such as reflux at the injection site, residual volume in the syringe, the needle gauge used, and the scleral incision^12^,^14^,^26^. Acute IOP elevation has been reported to potentially increase the risk of intraocular circulatory disorders^8^,^16^,^17^. Several studies have reported that the rapid increase IOP returned to normal value soon without IOP-lowering therapy following intravitreal injection. Here, we utilize an intravitreal infusion rabbit model to observe if intravitreal infusion prevents that rapid increase in IOP that occurs with intravitreal injections and the accompanying side-effects.

The results of our study suggest that the IOP remains relatively stable in groups A and B. As the drug doses increased in groups C and D, the IOP increased significantly and light to moderate corneal edema was observed. This suggests an efficient IOP homeostatic mechanism that aims to maintain a stable IOP when the volume is increased in the vitreous body. However, this can only occur at certain volumes and infusion speeds. Compared with group B, the post-injection IOP in group E and the post-infusion IOP in group F were significantly higher. It is currently accepted that intravitreal injections are safe, as the IOP typically returns to a safe range within 30 minutes^7^. However, an IOP measurement taken 30 minutes after application might miss the short-term increase for all eyes show a dramatic increase in IOP after injection. The values reached are remarkably higher than occlusion pressure for the central retinal artery^12^. Starting from 30 mm Hg, the retinal blood flow diminishes linearly with increasing IOP and is nearly stopped at 100 mm Hg. A reduction in optic nerve head capillary blood perfusion and choroidal perfusion at higher IOPs has also been observed^19^. Older eyes do not fully recover retinal function after acute IOP elevation, suggesting a more subtle age-related susceptibility to IOP^32^. Additionally, retinal structural measurements, such as surface displacement or tissue compression, are more sensitive to acute IOP elevation than electrophysiological measurements of retinal function^33^. Repeated acute IOP spikes may cause cumulative dysfunction and permanent, nonspecific damage in the retina^34^,^35^. Patients in need of vitreous injection are typically advanced in aged and require repeated treatments. Some of the patients have retinal vascular disorders or the underlying systemic disease. Repeated increased post-injection IOP may damage the retinal structure and function, and further impede ocular perfusion. Our study suggests that intraocular infusion has marked advantages in maintaining IOP stability and may reduce the risk factors associated with intravitreal injection. This may be particularly important for patients with a history of glaucoma and vascular diseases.

In our study, we measured the IOP values in group F after 100 μL were bolus infused into the vitreous cavity without the influence of reflux and residual volume. It showed that transient increase in IOP after the bolus infusion was greater than the post-injection IOP (group E), which suggests that the IOP was decreased in part by reflux and/or residual volume. As Benz *et al*. reported patients that did not experience vitreous reflux at the site of injection had a significant initial elevation in IOP. However, patients that experienced vitreous reflux at the site of injection had either no change in IOP or a small drop in IOP^15^. Additionally, residual drug remaining in the syringe was common after intravitreal injection^26^,^36^, which also occurred after intravitreal injection in our experiment. Deviations from the intended quantities in the amount of drug delivered to the eye could result in therapeutic failures^26^,^36^. In the infusion groups, IOP values at 30 minutes prior to removal of the needle and reflux scores had a significant statistical correlation. Thus, as the IOP was lower the amount of reflux was fewer. During the intravitreal infusion procedure, the last IOP measurement prior to removing the needle could be monitored and adjusted. Importantly, the accuracy of the amount of drug delivered is better controlled by using an infusion pump, as demonstrated in our experiments. Keith *et al*. compared the accuracy and precision of low-dose insulin administration using various devices. Their results suggested that pen and pump devices are more accurate, and specifically pump devices are more precise than syringes at 1- and 2-unit doses^37^. Overall, we conclude that intravitreal infusion using a pump device decreases reflux and improves the accuracy of drug dosage by controlling the IOP better than standard intravitreal injections.

In standard intravitreal injections the drug is placed into the vitreous cavity by the action of a syringe. A survey of retina specialists in the United States reported that a majority inject quickly among the 59% of participants who consider the speed of the jet of fluid they inject^1^. Peyman *et al*. recommend that injections should be slowly performed in order to avoid jet formation or cavitary flow^3^. In Aiello and associates’ guidelines for intravitreal injection it is recommended that injections be performed moderately slow as rapid injection causes excessive dispersion of the drug into the vitreous cavity^38^. However, currently there is no consensus in injection protocols about what is the appropriate perfusion speed. By contrast to injections, the pump device used in intravitreal infusions controls the speed and avoids the inaccuracies of manual manipulation. Future research should be performed to determine the appropriate infusion velocity, which would make great strides in the development of a standard intravitreal drug delivery procedure. Additionally, as infusion times could be lengthened to accommodate larger doses than injections, infusion could be applied to endophthalmitis and pneumatic retinopexy.

The biggest disadvantage of intravitreal infusion is the length of time the treatment takes to be performed. In our animal experiments intravitreal infusion was performed under general anesthesia. Performing this procedure in patients would require the patient to remain still for a prolonged period of time. It is unknown if longer infusion times will contribute to further complications, such as the development of endophthalmitis, cataract, retinal detachment, or corneal injury. In addition, surface anesthesia may not be enough and peribulbar anesthesia may be required.

As this is one of the first studies exploring the use of intravitreal infusions several limitations exist. First, the pump device used is specialized for diabetes and should be modified for intravitreal infusion. For example, the shortest infusion time using the diabetic insulin pump is 30 minutes and the needle size is 27-gauge. These parameters could be adjusted to better suit intravitreal infusions and suitable equipment should be designed specifically for intravitreal infusions. Second, the residual volume was roughly estimated using the scale of the syringe. More precise measurements would be obtained if each dose was weighed using an analytical scale. However, under the current setup, the syringe is connected to the pump and the infusion tube and it is not feasible to perform accurate measurements. Another limitation of our study is the differences between rabbit and human eyes. But the relevant anatomical and physiological parameters in rabbit and man show only small differences. The rabbit is the most commonly used animal model for intravitreal pharmacokinetic studies^39^.

In summary, our study demonstrates that intravitreal infusion maintains stable IOP and improves the accuracy of drug delivery compared to the standard intravitreal injection procedure. However, the procedure time is increased and potential complications are not fully understood. Overall, intravitreal infusion is very promising in the advancement of drug delivery to the posterior segment of the eye.

## Methods

### Infusion devices

Insulin pumps (Fornia, Zhuhai, Guangdong, China) were used as infusion devices. The device includes a pump (including controls, processing module and batteries), a disposable reservoir for insulin or drug (within the pump) and a disposable infusion set (including a needle for subcutaneous insertion and a tubing system to interface the insulin reservoir to the needle). All needles used were 27-gauge. Dosage instructions were programed into the device to ensure the amount of drug was injected in a calculated and controlled manner. Two infusion methods were used in the study. The first method was a slow infusion and the drugs were administered once per minute in equal doses over a 30 minute time period. The second method, most similar to injections, was a bolus infusion of drug pumped completely at the onset of the bolus at the rate of 10 μL/6 s.

### Animals

All procedures were performed in accordance with the ARVO Statement for Use of Animals in Ophthalmic and Vision Research and approved by the animal ethics committee of Central South University, Xiangya School of Medicine (approval ID: 2011-034). All surgeries were performed under sodium pentobarbital anesthesia. The study included 36 white New Zealand rabbits with an average weight of 2000 g and were housed and bred at a certified animal care facility at the Second Xiangya Hospital of Central South University. The animals were anesthetized by intravenous injection pentobarbital sodium (30 mg/kg, Sigma; St. Louis, MO, USA) and topical anesthesia was administered using oxybuprocain hydrochloride 0.4% eye drops. Pupils were dilated with a topical application of phenylephrine hydrochloride 0.5% and tropicamide 0.5% eye drops. Ofloxacin 0.3% eye drops were topically administered before and after treatment.

### Design of the study

TB 0.06% was used for intravitreal infusion or injection to enable visualization of the reflux at puncture site. A tunneled intravitreal puncture was performed at 2 mm posterior to the limbus through the pars plana located in the superior quadrant of the eye.

The experiment was designed with four groups with different volumes to evaluate the safety of infusion speed. Groups A (50 μl), B (100 μl), C (150 μl) and D (200 μl) were slowly infused over 30 minutes with TB using an insulin pump. Each group included six rabbits and infusions were done in the right eye. During the infusion process, the cotton pads wetted with physiological saline was used to cover the cornea.

In order to compare to standard intravitreal injections we created two additional experimental groups. According to the results group A–D, group B was set as the reference group. Group E (n = 6) underwent bolus injection of 100 μL of TB as usually done in clinical practice. Group F (n = 6) received a bolus infusion with 100 μL of TB within one minute using an infusion pump and the needle was withdrawn after 30 minutes.

Eyes were evaluated using the following parameters and possible complications were observed during the procedure and post-operation on day one, three, and seven. All procedures were performed by the same physician (Tian J).

### IOP measurement

IOP was measured using a Tono-pen XL tonometer (Reichert, Inc; Depew, NY, USA) at 0, 5, 10, 15, 20, 25, 30 minutes and post-operation (at about 31 minutes) in all intravitreal infusion groups. In the intravitreal injection group (group E) IOP was measured before and after injection. The tonometer was calibrated according to the manufacturer’s instructions prior to the experiment. Three values were recorded for each time point. Measurements were repeated until the coefficient of variation was less than 5%. The final results represent the median of three measurements. IOPs greater than 60 mmHg often result in high levels of fluctuation and were recorded as 60 mmHg.

### Measurement of Reflux

The TB reflux or vitreous incarceration was calculated by measuring the longest length of the reflux spot size visible at the entry site. Scores were assigned 0 to 3 and were used to describe the degree of reflux. If no reflux was observed at the needle insertion site it scored as 0. Reflux spots less than 2 mm were scored as a 1. Reflux spots 2 to 4 mm in diameter were scored as a 2 and spots more than 4 mm were scored as a 3.

### Residual volume

The programmable infusion pump controls the dosage of infusions and has a reported accuracy of ±0.5 μL. The same disposable drug reservoir can be used for several eyes by simply changing the infusion tube. Additionally, the volumes needed to purge air from the tube were recorded. In the infusion groups the distinction between the predicted residual volume and the actual remaining volume were assessed by the scale of the reservoir. In the injection group the residual volumes were assessed through the scale of the syringe.

### Statistical Analysis

Statistical analyses were performed using the statistical software package IBM SPSS Statistics version 19.0 (IBM Corp., Somers, NY, USA). MANOVA was used to compare the IOP values of groups A, B, C and D. The correlation of IOP values before removal of needle and the scores of reflux in all intravitreal infusion eyes were calculated using the Spearman’s rank correlation coefficient. Statistical significance was considered when *p* < 0.05.

## Additional Information

**How to cite this article**: Tian, J. *et al*. Intravitreal infusion: A novel approach for intraocular drug delivery. *Sci. Rep.* **6**, 37676; doi: 10.1038/srep37676 (2016).

**Publisher's note:** Springer Nature remains neutral with regard to jurisdictional claims in published maps and institutional affiliations.
